# Three Key Sub-leaf Modules and the Diversity of Leaf Designs

**DOI:** 10.3389/fpls.2017.01542

**Published:** 2017-09-06

**Authors:** Le Li, Zeqing Ma, Ülo Niinemets, Dali Guo

**Affiliations:** ^1^Center for Forest Ecosystem Studies and Qianyanzhou Ecological Station, Key Laboratory of Ecosystem Network Observation and Modeling, Institute of Geographic Sciences and Natural Resources Research, Chinese Academy of Sciences Beijing, China; ^2^College of Resources and Environment, University of Chinese Academy of Sciences Beijing, China; ^3^Department of Plant Physiology, Institute of Agricultural and Environmental Sciences, Estonian University of Life Sciences Tartu, Estonia; ^4^Estonian Academy of Sciences Tallinn, Estonia

**Keywords:** leaf anatomical structure, leaf diversity, leaf form, leaf function, stomata, venation

## Abstract

Earth harbors a highly diverse array of plant leaf forms. A well-known pattern linking diverse leaf forms with their photosynthetic function across species is the global leaf economics spectrum (LES). However, within homogeneous plant functional groups such as tropical woody angiosperms or temperate deciduous woody angiosperms, many species can share a similar position in the LES but differ in other vital leaf traits, and thus function differently under the given suite of environmental drivers. How diverse leaves differentiate from each other has yet to be fully explained. Here, we propose a new perspective for linking leaf structure and function by arguing that a leaf may be divided into three key sub-modules, the light capture module, the water-nutrient flow module and the gas exchange module. Each module consists of a set of leaf tissues corresponding to a certain resource acquisition function, and the combination and configuration of different modules may differ depending on overall leaf functioning in a given environment. This modularized-leaf perspective differs from the whole-leaf perspective used in leaf economics theory and may serve as a valuable tool for tracing the evolution of leaf form and function. This perspective also implies that the evolutionary direction of various leaf designs is not to optimize a single critical trait, but to optimize the combination of different traits to better adapt to the historical and current environments. Future studies examining how different modules are synchronized for overall leaf functioning should offer critical insights into the diversity of leaf designs worldwide.

## Introduction

As the metabolic engine of plant growth, leaves are marvelous examples of biological complexity and natural beauty (Vogel, 2012; Sack, 2013). Remarkable diversity occurs in the designs of leaf form and function. On what principles do diverse leaf forms and functions vary from one species to another? Comparative studies of leaf functional traits have been highly successful at identifying key trade-offs in leaf form and function (Givnish, 1987; Westoby et al., 2002; Ackerly, 2004). Among the numerous successes, the most noteworthy is probably the global leaf economics spectrum (LES), which states that a universal trade-off exists between leaf construction costs [characterized by leaf dry mass per area (LMA)] and leaf lifespan (LL) across different biomes and contrasting plant functional groups (Wright et al., 2004). This whole-leaf level cost-benefit perspective has been valuable for understanding leaf carbon balance as well as whole plant growth (Poorter et al., 2013; Westoby et al., 2013; Reich, 2014).

Although highly successful, the economics spectrum may be insufficient for a full understanding of the complex evolutionary steps and adaptation to current abiotic and biotic environments in leaves as well as in whole plants. Emerging studies have revealed that leaves with similar LMA can differ in other critical traits such as leaf hydraulics (Sack et al., 2014; Li et al., 2015; Blackman et al., 2016), stomatal conductance (Reich et al., 1999) and leaf drought tolerance (Maréchaux et al., 2015) as well as whole plant drought and shade tolerances (Hallik et al., 2009). These studies suggest that different species do not always spread out widely along a single axis of carbon economy (i.e., LMA-LL); rather, they may vary along multiple dimensions of trait trade-offs. In fact, only through the lens of multiple dimensions of leaf traits and corresponding ecological and physiological functions, the immense diversity of leaf designs observed in the plant kingdom does seem possible (Laughlin, 2014; Li et al., 2015; Blackman et al., 2016).

**FIGURE 1:**
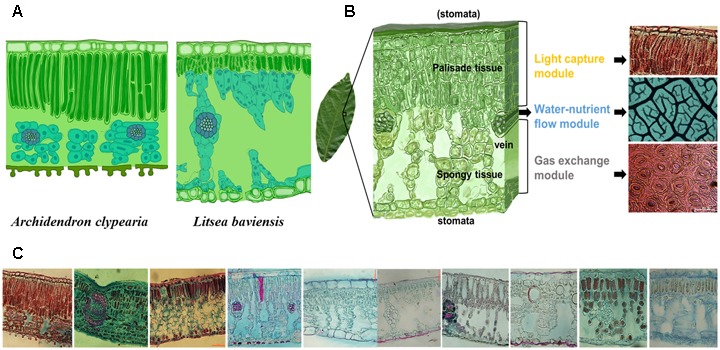
Illustration of the concept of modularity of leaf design. Different species can have different internal leaf structures such that the key leaf trait, leaf dry mass per unit area (LMA) alone may not be informative enough for describing the diversity of leaf designs as exemplified by a comparison of two species with similar LMA (77.3 g m^-2^ for *Archidendron clypearia* and 68.9 g m^-2^ for *Litsea baviensis*) **(A)**. A frequently observed mode for the combination of three key sub-leaf modules: from top to bottom, a leaf can be divided into a light capture module, a water-nutrient flow module and a gas exchange module **(B)**. Demonstration of the vast diversity of leaf internal structures. Such diverse leaf designs can be seen as the result of different adjustments to the frequently-observed mode **(C)**.

To fully understand the multi-dimensionality of leaf form and function, a whole leaf needs to be divided into different component parts, each responsible for a specific function. Indeed, LMA is a product of leaf thickness and tissue density (Niinemets, 1999; Poorter et al., 2009), both of which are influenced by anatomical drivers such as leaf cellular and tissue composition, airspace volume and structure, and ultrastructural cell characteristics (Villar et al., 2013; John et al., 2017). Different combinations of leaf anatomical traits can result in similar LMA values across species (**Figure 1A**), but different values of these traits affect leaf-level water transport (Tyree et al., 1999; Sack et al., 2003; Sack and Holbrook, 2006; Brodribb, 2015; Buckley et al., 2015), CO_2_ diffusion (Terashima et al., 2011; Muir et al., 2016), and light capture (Vogelmann et al., 1996) differently. Therefore, a whole leaf analysis alone might hide the linkages between the key structural components and their corresponding functions. For a fully functional analysis, it can be illuminating to employ the concept of modularity, which implies that different independent (or semi-independent) modules each carrying a different function can be integrated as a whole (Wagner and Altenberg, 1996).

Here, we propose that a leaf can be divided into three key modules, and each module is composed of a set of correlated leaf traits. We emphasize that different modules are not necessarily fully independent from each other, but identifying each module can serve as a tool for tracing the evolutionary pathways of key leaf physiognomies and functions. We also argue that a leaf is an evolutionary patchwork composed of separate modules and that deciphering different trait combinations of the modules will offer new insights into the diverse leaf structures and functioning.

## Three Key Sub-Leaf Modules

**FIGURE 2:**
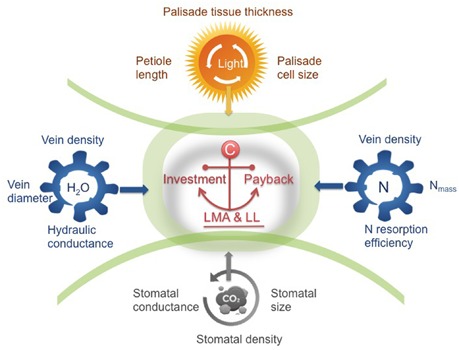
A trait framework based on the three key sub-leaf modules. In the center, the leaf economics spectrum (LES) concept of integrating investment and payback (LMA and leaf longevity, LL) is a fundamental principle, defining the trade-offs of the three modules. By unpacking the trade-off between LMA-LL into four other relations (i.e., interactions between leaves and light-water-nutrients-gas composition), we show the pathway moving beyond traditional leaf economics. Taking this framework as a starting point, we can have a more holistic understanding of global-scale variation in leaf structure and function. Certain trait syndromes are suggested for each module, ranging from cell size, tissue-level density or thickness to the rates of physiological processes.

A leaf is highly heterogeneous in its structure and can be divided into three major types of modules corresponding to light capture, water-nutrient transport, and CO_2_ absorption. The first module is the light capture module, which is mainly composed of epidermal cells and tightly packed palisade cells (**Figure 1B**). The epidermal cells are transparent and do not contain chlorophyll, however, the outer cell wall can function as a lens focusing light deeper into the leaf interior (Vogelmann and Björn, 1986; Martin et al., 1989; Poulson and Vogelmann, 1990). Furthermore, palisade cells can operate as optical fibers, allowing the penetration of light deeper into the leaf (Vogelmann and Martin, 1993; Smith et al., 1997; Brodersen et al., 2008). Nevertheless, due to high concentration of chlorophyll, palisade cells are responsible for the majority of light capture (Lee et al., 1990; Vogelmann et al., 1996). Besides, leaf petioles and midribs serve to determine leaf angle (Niinemets et al., 2006, 2007b), which further can modulate light interception (Salisbury, 1949; Smith et al., 1998; Falster and Westoby, 2003; de Boer et al., 2016). Apart from these main components, some plant species can have unique features such as vertical sclereids that can also function as optical fibers for light penetration (Karabourniotis, 1998; Nikolopoulos et al., 2002). Typically, the light capture module is primarily associated with the upper layer of a leaf, especially for horizontally inclined leaves (Parkhurst and Givnish, 1986; Smith et al., 1997). In particular, in dense canopies (e.g., forests and woodlands as well as dicot herb canopies), most leaves receive light from high solar inclination angles; once the light enters the leaf, light intensity decreases rapidly (Vogelmann et al., 1996). Some key trait characteristics to this module include epidermal outer cell wall properties, palisade cell size, palisade tissue thickness, chlorophyll content, and petiole length (**Figure 2**).

The second module is the water-nutrient flow module, which is mainly composed of leaf venation network (**Figure 1B**). The leaf venation network consists of both major and minor veins, with major veins being responsible for transport and partitioning of water and nutrients hierarchically throughout different orders of leaf venation system, and minor veins being responsible for delivering the water to precise locations to meet the water demands for photosynthesis and water transpiration through the stomata (Roth-Nebelsick et al., 2001; Sack and Holbrook, 2006; Brodribb et al., 2007; Sack and Scoffoni, 2013). Furthermore, leaf venation provides an indispensable network for the flow of nutrients and other materials throughout the leaf and to outside the leaf via phloem and xylem, including nitrogen resorption (Zhang et al., 2015) and export of photosynthetic products (Turgeon, 2006). Some key traits involved in water-nutrient flow module include vein diameter and density, vessel (or tracheid) size and density, leaf hydraulic conductance and nitrogen resorption efficiency (**Figure 2**).

The third module is the gas exchange module, which is mainly composed of stomata and connected intercellular air spaces in the palisade and spongy mesophyll and mesophyll cells with chloroplasts (**Figure 1B**). Stomata are sensitive valves for CO_2_- H_2_O exchange and can respond rapidly to plant water availability and evaporative demand, which is influenced by air humidity and leaf temperature as affected by ambient temperature and leaf radiation interception and loss (Smith et al., 1997; Hetherington and Woodward, 2003; Franks et al., 2009). In addition, chloroplast characteristics and cell position alter the gradients of CO_2_ diffusion from sub-stomatal cavities to chloroplasts (Flexas et al., 2012; Tomás et al., 2013; Tosens et al., 2016). Variation of stomatal structure, such as stomatal crypts and waxy deposits can also affect leaf gas exchange (Roth-Nebelsick, 2007). Some key traits involved in the gas exchange module include stomatal size, stomatal density, stomatal conductance, the size and distribution of chloroplasts with respect to cell walls and exposure of cell walls to the gas phase inside the leaf, and photosynthetic capacity per cell (**Figure 2**).

## Evolutionary History for Each Sub-Leaf Module

In understanding the current diversity across these three sub-modules, it is important to consider that the evolution of different leaf modules was often largely independent, i.e., key features of each module evolved in response to their most prominent selection pressures. The light capture module underwent substantial selection as plants radiated to more open habitats (Feild and Arens, 2005), the water-nutrient flow module was modified by ever increasing transpirational pull in more open habitats (Feild et al., 2003), and the gas exchange module was modified in response to the gradually declining atmospheric CO_2_ concentration (Franks and Beerling, 2009). Over time, different configurations of these modules were combined into various leaves. The evolutionary direction of various leaf designs is not to optimize a single critical trait or module, but to optimize the combination of different traits to better adapt to the highly variable historical and extant environments that diverse species across different plant types occupy.

In particular, for angiosperms which constitute one of the most important plant groups in terms of species diversity and biomass production on the Earth, the 400-million-year history is also a step-by-step process. Fewer trait combinations among a limited number of early angiosperm species diversified into more trait combinations among a high number of derived angiosperm species. During this process, gaining new advanced functions and capacities was a key mechanism for angiosperm radiation and diversification. Compared to other plant groups, the underlying superiority of angiosperms in having more diverse trait combinations is also confirmed by recent global synthesis of leaf traits (see Diaz et al., 2016).

During early angiosperm evolution, the light capture module was rather weakly developed among woody angiosperms (i.e., during the early Cretaceous *c.* 135 Ma) (Bond and Scott, 2010). Many ancestral species, such as *Amborella*, had little or no differentiation of palisade mesophyll from spongy mesophyll (Feild et al., 2003; Feild and Arens, 2005). Such a weakly developed light capture module was widespread in the understory shady habitats (Feild and Arens, 2005; Bond and Scott, 2010). Yet, as more woody angiosperms radiated into open habitats, a more clearly differentiated light capture module began to appear, especially among canopy angiosperms. These species shared the common characteristic of multiple layers of tightly packed palisade tissue.

The evolution of water-nutrient flow module has also been a step by step process. One notable aspect of this gradual process is the substantial increase of vein density in angiosperms compared to other plant groups (Boyce et al., 2009; Feild et al., 2011a), which took about 250 million years. High leaf vein densities endowed woody angiosperms with higher leaf water supply capacity than that in their competitors (Boyce et al., 2009), and contributed to the rise of photosynthetic capacity in the early angiosperm diversification (Brodribb and Feild, 2010), but nevertheless, there are still large differences in leaf vein density within this group (Boyce et al., 2009). Other concurrent innovations include the decreasing size of vein diameter (Feild and Brodribb, 2013), the unique spatial arrangements of veins (Zwieniecki and Boyce, 2014), the modifications of perforation plates of primary xylem vessels (Feild and Brodribb, 2013), and the reduction of cell wall thickness and chloroplast size improving the rate of CO_2_ diffusion into chloroplasts (Tosens et al., 2016; Veromann et al., 2017), all these traits showing higher overall values than more ancient plant groups but large differences within the woody angiosperm groups.

Concurrently with the decline in atmospheric CO_2_ concentration, innovations of the gas exchange module took place gradually over the past 400 million years. As atmospheric CO_2_ concentration decreased, stomatal density increased, while stomatal size decreased among most land plants. The increases in stomatal density contributed to an overall increase in the maximum stomatal conductance, while decreased stomatal size enabled greater sensitivity to environmental changes (Franks and Beerling, 2009). In the early Cretaceous, early angiosperm leaves had a low gas exchange capacity with a low maximum stomatal pore area (Feild et al., 2011b). The overall superior performance of angiosperms was achieved by gaining the capacity to close stomata in response to high CO_2_ concentration, ensuring a higher water use efficiency than that in other lower land plants (Brodribb et al., 2009), but again, with high variations across species within this group.

## Diversity of Leaf Designs

An important goal of this paper is to highlight how a modularized-leaf perspective may provide a useful approach for characterizing and understanding the variation of leaf form and function beyond traditional LES perspective (**Figure 1C**). LES mainly defines the large-scale leaf design patterns across different leaf types and different biomes, while our concepts of key sub-leaf modules can further describe the variation of smaller-scale leaf designs within one leaf type or/and one plant functional type (PFT) or/and one biome. Though LES has a great value in revealing global-scale leaf strategies (Diaz et al., 2016), it is insufficient for describing the diversity of leaf designs. A global meta-analysis of variation of LMA demonstrated that LMA failed to distinguish between species within a biome or a functional group (Poorter et al., 2009). For example, within species-rich subtropical forests, *Pithecellobium clypearia* and *Litsea baviensis* (77.3 and 68.9 g m^-2^) are both evergreen woody angiosperms species with similar LMA, but they can differ markedly in leaf light-capture and water-nutrient flow modules (**Figure 1A**). As already argued, different species can differentiate their leaves by modifying module-level traits without altering the values of LMA (**Figure 2**). Ultimately, the divergent modular construction of leaves underlies the emergent trade-offs between leaf structures and function known as the worldwide LES (Wright et al., 2004).

In fact, different sub-leaf modules are not necessarily fully independent from each other, and plenty of studies have shown coordinated variation across modules (e.g., Sack et al., 2003; Brodribb et al., 2005, 2007). Given that different modules develop together, they might often be interdependent. Especially, when there is a limiting factor (e.g., light, nutrients, or water), these three sub-leaf modules would converge toward maximizing the use of the limiting resource (Mason and Donovan, 2015). Nonetheless, even though different modules co-vary, the direction of such covariations is not fixed. For instance, leaf nitrogen concentration can be either positively (Schulze et al., 1994), or negatively correlated (Taylor and Eamus, 2008) with maximum stomatal conductance.

Even more importantly, under non-constraining growth conditions, key traits corresponding to each module *can* have room to evolve independently and maximize each function separately without constrains limiting these maxima. For example, leaf carbon and water use related traits are independent among 85 woody angiosperms in tropical-subtropical forests (Li et al., 2015), and vein density (water-nutrient flow module) was unrelated to stomatal conductance (gas-exchange module) among 35 evergreen Australian angiosperms (Gleason et al., 2016). Such independence across modules can lead to diverse combinations of different traits, and consequently to the high diversity of leaf designs in species-rich biomes. Indeed, leaves do contain different fractions of mesophyll, epidermis and vascular tissues, and these fractions always adjust to different environmental conditions, with different environmental pressures altering the distribution of leaf tissues in different manners (Niinemets, 1999; Niinemets et al., 2007a). In fact, if sub-leaf modules always co-varied and did so in a consistent manner, possible combinations of leaf structures across species would be very few, leaving relatively little room for adaptation to different environments.

Although we often find these three modules arranged in the sequence shown in **Figure 1B**, spatial arrangements of these sub-leaf modules can vary across different habitats and different plant types (e.g., angiosperms, gymnosperms, ferns, mosses, and aquatic plants). Variation in modular arrangement can lead to different configurations of these three key modules, and consequently to even greater diversity of leaf designs. Stomata can develop on both surfaces of a leaf, termed as amphistomatous leaves (Parkhurst, 1978), which is a derived feature for angiosperms (Mott et al., 1982). Amphistomatous species with high conductance to CO_2_ diffusion (Mott et al., 1982; Beerling and Kelly, 1996; Muir, 2015) are most common among fast-growing species (Muir, 2015), and are usually successful in high-light habitats (Mott et al., 1982; Beerling and Kelly, 1996; Muir, 2015). Similarly, palisade cells can also be found on both the lower and upper layers of a leaf (Smith et al., 1997). Examples can be found in the isobilateral leaves of eucalypts in arid environments (Evans et al., 1993; Ögren and Evans, 1993; de Boer et al., 2016), and in the trees of the open savannas in the Amazon (e.g., *Byrsonima crassifolia*) (Ferreira et al., 2015).

## Future Directions

From the perspective of leaf modularity, studies of leaf functional traits and leaf diversity are approaching a new era. Recognizing the corresponding trait syndromes for each module, rather than focusing only on whole-leaf traits, is strongly recommended for future leaf trait-based studies attempting to understand the mechanisms of species diversity and species-environment relationships (**Figure 2**). The LES concept of integrating investment and payback (LMA-LL) is a fundamental principle, defining the trade-offs of the three modules. Thus, variation in LMA is a by-product of individual responses of different modules to the environment (**Figure 2**). By considering different components of LMA in relation to four key environmental drivers (i.e., light, water, and nutrient availabilities, and gas composition) (**Figure 2**), and examining how different modules interact with the environment, we can obtain a more mechanistic understanding of patterns of leaf structure and function at both local and global scales. Besides, to improve predictions of species responses to environmental changes, more attention should be paid to the environmental response curves of each module in future predictive models of global change that intend to use leaf traits to predict future vegetation.

Another important step forward will be to examine trait combinations of different sub-leaf modules across species at the global scale. In this way, we can pinpoint the underlying mechanisms driving different ecological strategies and high leaf diversity. Under such a framework, both significant and non-significant correlations among different leaf traits can be useful for identifying coordination and independence both within and among different modules. Furthermore, we can trace back how different modules were integrated into a single leaf over time among different plant functional groups having different leaf types. In future biodiversity models, detailed leaf functional types (LFTs) can be defined for better representation of leaf diversity, by identifying different combinations of sub-leaf modules both within one PFT and across different PFTs. Overall, combinations of different modules have contributed to different leaf ecological strategies and thus, to tremendous plant diversity over the long evolutionary history, and will continue to shape leaf and whole-plant responses in the future.

## Author Contributions

LL and DG developed the idea. LL prepared the first draft of the manuscript. All authors made substantial contributions to the revision of the manuscript.

## Conflict of Interest Statement

The authors declare that the research was conducted in the absence of any commercial or financial relationships that could be construed as a potential conflict of interest.
